# 4-Hy­droxy-6-(4-meth­oxy­phen­yl)-4-phenyl-1,3-diazinane-2-thione

**DOI:** 10.1107/S1600536811008002

**Published:** 2011-03-09

**Authors:** H. C. Devarajegowda, K. R. Roopashree, Irfan Ali Mohammed, Mahalakshmi, Ravish Sankolli

**Affiliations:** a Department of Physics, Yuvaraja’s College (Constituent College), University of Mysore, Mysore 570 005, Karnataka, India; b Department of Pharmaceutical Chemistry, Sri Adichunchangiri College of Pharmacy, B.G. Nagar 571 448, Mandya, Karnataka, India; c Solid State and Structural Chemistry Unit, Indian Institute of Science, Bangalore 12, Karnataka, India

## Abstract

In the title compound, C_17_H_18_N_2_O_2_S, the 1,3-diazinane-2-thione ring system is not coplanar with the benzene ring and meth­oxy­phenyl ring system, the dihedral angle between the planes being 65.58 (13) and 89.18 (10)°, respectively. The crystal structure is characterized by inter­molecular O—H⋯S, N—H⋯S, N—H⋯O and C—H⋯S hydrogen bonding.

## Related literature

For general background to pyrimidines, see: Cheng (1969 ▶); Scott *et al.* (1959 ▶); Jonak *et al.* (1972 ▶); Falco *et al.* (1961 ▶); Ram (1990 ▶); Howells *et al.* (1981 ▶); Pershin *et al.* (1972 ▶); Matolcsy (1971 ▶); Prikazchikova *et al.* (1975 ▶). For the synthesis, see: Paghdar *et al.* (2007 ▶). For a related structure, see: Yamin *et al.* (2005 ▶).
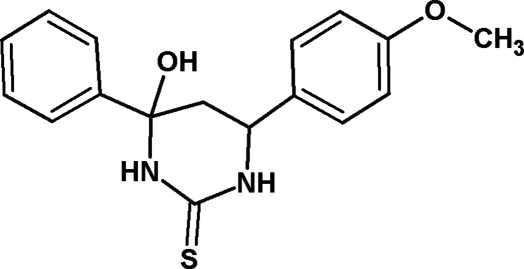


## Experimental

### 

#### Crystal data

C_17_H_18_N_2_O_2_S
*M* *_r_* = 314.39
Monoclinic, 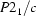

*a* = 12.6016 (3) Å
*b* = 6.3375 (1) Å
*c* = 20.6637 (4) Å
β = 97.890 (2)°
*V* = 1634.64 (6) Å^3^
*Z* = 4
Mo *K*α radiation
μ = 0.21 mm^−1^
*T* = 295 K
0.18 × 0.16 × 0.16 mm

#### Data collection

Oxford Diffraction Xcalibur diffractometer
Absorption correction: multi-scan (*CrysAlis PRO RED*; Oxford Diffraction, 2010 ▶) *T*_min_ = 0.963, *T*_max_ = 1.000
18135 measured reflections
3547 independent reflections
2566 reflections with *I* > 2σ(*I*)
*R*_int_ = 0.039

#### Refinement

*R*[*F* ^2^ > 2σ(*F* ^2^)] = 0.045
*wR*(*F* ^2^) = 0.115
*S* = 1.08
3547 reflections
215 parameters
H atoms treated by a mixture of independent and constrained refinement
Δρ_max_ = 0.24 e Å^−3^
Δρ_min_ = −0.17 e Å^−3^

### 

Data collection: *CrysAlis PRO CCD* (Oxford Diffraction, 2010 ▶); cell refinement: *CrysAlis PRO CCD*; data reduction: *CrysAlis PRO RED* (Oxford Diffraction, 2010 ▶); program(s) used to solve structure: *SHELXS97* (Sheldrick, 2008 ▶); program(s) used to refine structure: *SHELXL97* (Sheldrick, 2008 ▶); molecular graphics: *ORTEP-3* (Farrugia, 1997 ▶) and *CAMERON* (Watkin *et al.*, 1993 ▶); software used to prepare material for publication: *WinGX* (Farrugia, 1999 ▶).

## Supplementary Material

Crystal structure: contains datablocks I, global. DOI: 10.1107/S1600536811008002/bv2177sup1.cif

Structure factors: contains datablocks I. DOI: 10.1107/S1600536811008002/bv2177Isup2.hkl

Additional supplementary materials:  crystallographic information; 3D view; checkCIF report

## Figures and Tables

**Table 1:** Hydrogen-bond geometry (Å, °)

*D*—H⋯*A*	*D*—H	H⋯*A*	*D*⋯*A*	*D*—H⋯*A*
O2—H2⋯S1^i^	0.88 (3)	2.51 (3)	3.3688 (18)	169 (2)
C14—H14⋯S1^i^	1.030 (19)	2.695 (18)	3.666 (2)	157.0 (14)
N4—H4⋯S1^ii^	0.86	2.47	3.2990 (19)	163
N5—H5⋯O3^iii^	0.86	2.18	3.032 (2)	169
C20—H20⋯S1^iv^	0.93	2.86	3.772 (2)	166

Symmetry codes: (i) 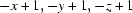; (ii) 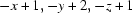; (iii) 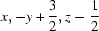; (iv) 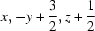.

**Fig. 1.**
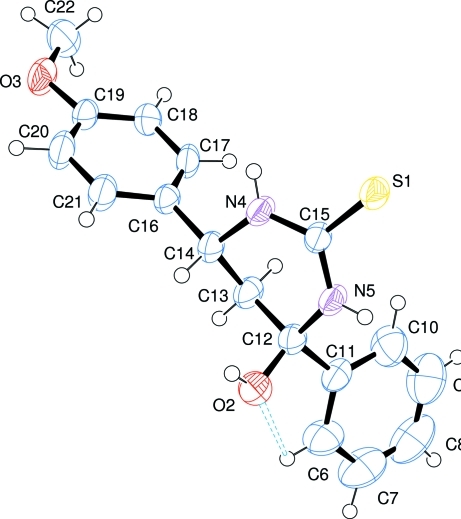
The molecular structure of the title compound. Displacement ellipsoids are drawn at the 50% probability level. Dashed lines indicate hydrogen bonds

**Fig. 2.**
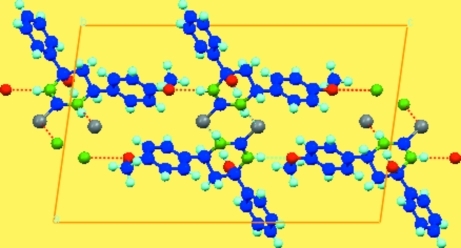
A packing view of the structure down the axis b.

## supplementary crystallographic information

### Comment

Pyrimidines, being an integral part of DNA and RNA, exhibit diverse pharmacological properties as effective bactericide, fungicide, viricide, insecticide, and medicinal agents (Cheng, 1969; Scott *et al.*, 1959). Certain pyrimidines and annulated pyrimidine derivatives are also known to display anticancer, antimalarial, antileishmanial and antifilarial activities (Jonak *et al.*, 1972; Falco *et al.* 1961; Ram, 1990; Howells *et al.*, 1981). Pyrimidines and thio-pyrimidines play an essential role in several biological processes and have a considerable chemical and pharmacological importance. In particular, the pyrimidine nucleus can be found in a broad variety of antibacterial and antitumor agents as well as in agrochemical and veterinary products (Pershin *et al.*, 1972; Matolcsy, 1971; Prikazchikova *et al.*, 1975).

The asymmetric unit of the 4-hydroxy-6-(4-methoxyphenyl)-4-phenyl tetrahydropyrimidine-2(1*H*)-thione contains one molecule (Fig. 1). The thio-tetrahydropyrimidine ring system is not coplanar with the benzene ring and methoxyphenyl ring system; the dihedral angle between the two planes 65.58 (13)° and 89.18 (10)° respectively. The crystal structure shows intermolecular O2—H2···S1, N4—H4···S1, N5—H5···O3, C14—H14···S1 & C20—H20···S1 and C6—H6···S1 intramolecular hydrogen bonds. Bond distances and bond angles within the aromatic rings are in agreement with those observed in a related structure (Yamin *et al.*, 2005).

### Experimental

A general procedure for the synthesis of 4-hydroxy-6-(4-methoxyphenyl)- 4-phenyl-3,4-dihydropyrimidine-2(1*H*)-thione is given in Paghdar *et al.*, 2007. An equimolar mixture of (2E)-1,3-diphenylprop-2-en-1-one and thiourea (0.01 mol) were dissolved in minimum amount of ethanol. Potassium hydroxide solution (2.5 ml) was added slowly and the mixture stirred for 10 h until the entire mixture becomes very cloudy. Then the mixture was neutralized with 10% HCl and poured slowly into 100 ml of cold water with constant stirring. The precipitate obtained was filtered, washed and recrystallized from ethanol.

### Refinement

All H atoms were positioned at calculated positions with O—H = 0.88 Å, N—H = 0.86 Å, C—H = 0.93 Å for aromatic H and 0.96 Å for methyl H and refined a riding model *U_iso_(H)* = 1.5*U_eq_(C)* for methyl H and 1.2*U_eq_(C)* for other and also refined independently fixing C14 with C—H = 0.1.036 Å and C13 with C—H = 0.96 Å & 0.99 Å respectively.

### Crystal data

**Table:**

C_17_H_18_N_2_O_2_S	*F*(000) = 664
*M**_r_* = 314.39	*D*_x_ = 1.278 Mg m^−^^3^
Monoclinic, *P*2_1_/*c*	Melting point: 392 K
Hall symbol: -P 2ybc	Mo *K*α radiation, λ = 0.71073 Å
*a* = 12.6016 (3) Å	Cell parameters from 3547 reflections
*b* = 6.3375 (1) Å	θ = 2.4–27.0°
*c* = 20.6637 (4) Å	µ = 0.21 mm^−^^1^
β = 97.890 (2)°	*T* = 295 K
*V* = 1634.64 (6) Å^3^	Plate, colourless
*Z* = 4	0.18 × 0.16 × 0.16 mm

### Data collection

**Table:**

Oxford Diffraction Xcalibur diffractometer	3547 independent reflections
Radiation source: Enhance (Mo) X-ray Source	2566 reflections with *I* > 2σ(*I*)
graphite	*R*_int_ = 0.039
Detector resolution: 16.0839 pixels mm^-1^	θ_max_ = 27.0°, θ_min_ = 2.4°
ω scans	*h* = −16→16
Absorption correction: multi-scan (*CrysAlis PRO RED*; Oxford Diffraction, 2010)	*k* = −8→7
*T*_min_ = 0.963, *T*_max_ = 1.000	*l* = −26→26
18135 measured reflections	

### Refinement

**Table:**

Refinement on *F*^2^	Primary atom site location: structure-invariant direct methods
Least-squares matrix: full	Secondary atom site location: difference Fourier map
*R*[*F*^2^ > 2σ(*F*^2^)] = 0.045	Hydrogen site location: inferred from neighbouring sites
*wR*(*F*^2^) = 0.115	H atoms treated by a mixture of independent and constrained refinement
*S* = 1.08	*w* = 1/[σ^2^(*F*_o_^2^) + (0.0447*P*)^2^ + 0.3347*P*] where *P* = (*F*_o_^2^ + 2*F*_c_^2^)/3
3547 reflections	(Δ/σ)_max_ < 0.001
215 parameters	Δρ_max_ = 0.24 e Å^−^^3^
0 restraints	Δρ_min_ = −0.17 e Å^−^^3^

### Special details

**Table:**

Experimental. *CrysAlis PRO*, Oxford Diffraction Ltd., Version 1.171.33.55 (release 05–01–2010 CrysAlis171. NET) Empirical absorption correction using spherical harmonics, implemented in SCALE3 ABSPACK scaling algorithm.
Geometry. All s.u.'s (except the s.u. in the dihedral angle between two l.s. planes) are estimated using the full covariance matrix. The cell s.u.'s are taken into account individually in the estimation of s.u.'s in distances, angles and torsion angles; correlations between s.u.'s in cell parameters are only used when they are defined by crystal symmetry. An approximate (isotropic) treatment of cell s.u.'s is used for estimating s.u.'s involving l.s. planes.
Refinement. Refinement of *F*^2^ against ALL reflections. The weighted *R*-factor *wR* and goodness of fit *S* are based on *F*^2^, conventional *R*-factors *R* are based on *F*, with *F* set to zero for negative *F*^2^. The threshold expression of *F*^2^ > 2σ(*F*^2^) is used only for calculating *R*-factors(gt) *etc*. and is not relevant to the choice of reflections for refinement. *R*-factors based on *F*^2^ are statistically about twice as large as those based on *F*, and *R*- factors based on ALL data will be even larger.

### Fractional atomic coordinates and isotropic or equivalent isotropic displacement parameters (Å^2^)

**Table:**

	*x*	*y*	*z*	*U*_iso_*/*U*_eq_	
S1	0.48694 (4)	0.81337 (8)	0.41473 (2)	0.04465 (17)	
O2	0.28051 (14)	0.2485 (2)	0.48527 (8)	0.0550 (4)	
O3	0.32640 (13)	1.1217 (2)	0.79728 (7)	0.0595 (4)	
N4	0.40048 (13)	0.7486 (3)	0.52278 (7)	0.0431 (4)	
H4	0.4430	0.8470	0.5390	0.052*	
N5	0.32830 (12)	0.5582 (2)	0.43340 (7)	0.0430 (4)	
H5	0.3310	0.5243	0.3934	0.052*	
C6	0.08485 (19)	0.2493 (4)	0.41656 (13)	0.0669 (7)	
H6	0.1074	0.1390	0.4448	0.080*	
C7	−0.0087 (2)	0.2288 (5)	0.37313 (17)	0.0873 (9)	
H7	−0.0479	0.1044	0.3725	0.105*	
C8	−0.0434 (2)	0.3852 (7)	0.33198 (16)	0.0943 (10)	
H8	−0.1061	0.3690	0.3029	0.113*	
C9	0.0139 (2)	0.5698 (6)	0.33308 (15)	0.0935 (9)	
H9	−0.0101	0.6793	0.3049	0.112*	
C10	0.1081 (2)	0.5928 (4)	0.37639 (13)	0.0715 (7)	
H10	0.1466	0.7182	0.3770	0.086*	
C11	0.14488 (16)	0.4324 (3)	0.41824 (10)	0.0464 (5)	
C12	0.24682 (15)	0.4557 (3)	0.46603 (9)	0.0423 (5)	
C13	0.22823 (17)	0.5908 (4)	0.52483 (10)	0.0474 (5)	
C14	0.33350 (16)	0.6441 (3)	0.56604 (9)	0.0421 (5)	
C15	0.39945 (14)	0.7009 (3)	0.46044 (9)	0.0369 (4)	
C16	0.32722 (15)	0.7792 (3)	0.62573 (9)	0.0397 (4)	
C17	0.27293 (16)	0.9682 (3)	0.62387 (9)	0.0454 (5)	
H17	0.2372	1.0162	0.5842	0.055*	
C18	0.27031 (16)	1.0888 (3)	0.67978 (9)	0.0456 (5)	
H18	0.2329	1.2157	0.6776	0.055*	
C19	0.32394 (16)	1.0182 (3)	0.73856 (9)	0.0442 (5)	
C20	0.38044 (19)	0.8316 (4)	0.74086 (10)	0.0568 (6)	
H20	0.4175	0.7849	0.7803	0.068*	
C21	0.38211 (18)	0.7138 (3)	0.68487 (10)	0.0521 (5)	
H21	0.4208	0.5885	0.6869	0.063*	
C22	0.2770 (3)	1.3220 (4)	0.79812 (12)	0.0780 (8)	
H22A	0.2851	1.3747	0.8421	0.117*	
H22B	0.2022	1.3093	0.7818	0.117*	
H22C	0.3101	1.4179	0.7710	0.117*	
H2	0.345 (2)	0.244 (4)	0.5073 (14)	0.081 (9)*	
H13A	0.1920 (17)	0.726 (3)	0.5100 (10)	0.054 (6)*	
H13B	0.1841 (16)	0.512 (3)	0.5504 (10)	0.054 (6)*	
H14	0.3700 (14)	0.504 (3)	0.5815 (9)	0.045 (5)*	

### Atomic displacement parameters (Å^2^)

**Table:**

	*U*^11^	*U*^22^	*U*^33^	*U*^12^	*U*^13^	*U*^23^
S1	0.0494 (3)	0.0552 (3)	0.0309 (3)	−0.0148 (2)	0.0107 (2)	−0.0048 (2)
O2	0.0585 (10)	0.0531 (9)	0.0499 (9)	−0.0108 (8)	−0.0050 (8)	0.0045 (7)
O3	0.0839 (11)	0.0644 (10)	0.0302 (7)	0.0027 (8)	0.0081 (7)	−0.0087 (7)
N4	0.0473 (9)	0.0555 (10)	0.0274 (8)	−0.0184 (8)	0.0085 (7)	−0.0080 (7)
N5	0.0476 (9)	0.0550 (10)	0.0273 (8)	−0.0169 (8)	0.0091 (7)	−0.0080 (7)
C6	0.0521 (14)	0.0733 (16)	0.0741 (17)	−0.0193 (12)	0.0038 (12)	−0.0066 (13)
C7	0.0505 (16)	0.109 (2)	0.099 (2)	−0.0249 (16)	−0.0020 (16)	−0.028 (2)
C8	0.0495 (16)	0.148 (3)	0.079 (2)	−0.002 (2)	−0.0135 (14)	−0.032 (2)
C9	0.0699 (19)	0.129 (3)	0.074 (2)	0.0122 (19)	−0.0166 (15)	0.0102 (19)
C10	0.0617 (15)	0.0838 (17)	0.0648 (17)	−0.0053 (14)	−0.0065 (13)	0.0077 (14)
C11	0.0424 (11)	0.0614 (13)	0.0356 (10)	−0.0074 (10)	0.0068 (9)	−0.0083 (9)
C12	0.0442 (11)	0.0490 (11)	0.0338 (10)	−0.0113 (9)	0.0061 (8)	−0.0011 (9)
C13	0.0492 (12)	0.0613 (14)	0.0335 (11)	−0.0173 (11)	0.0122 (9)	−0.0069 (10)
C14	0.0493 (11)	0.0496 (12)	0.0278 (10)	−0.0084 (10)	0.0072 (8)	−0.0008 (8)
C15	0.0373 (10)	0.0436 (10)	0.0293 (9)	−0.0026 (8)	0.0028 (8)	−0.0008 (8)
C16	0.0418 (10)	0.0504 (11)	0.0276 (9)	−0.0110 (9)	0.0076 (8)	−0.0007 (8)
C17	0.0509 (12)	0.0593 (13)	0.0251 (9)	−0.0048 (10)	0.0015 (8)	0.0054 (9)
C18	0.0528 (12)	0.0487 (11)	0.0362 (11)	−0.0010 (10)	0.0093 (9)	0.0011 (9)
C19	0.0541 (12)	0.0522 (12)	0.0273 (9)	−0.0091 (10)	0.0091 (9)	−0.0020 (8)
C20	0.0732 (15)	0.0646 (14)	0.0294 (11)	0.0062 (12)	−0.0038 (10)	0.0006 (10)
C21	0.0633 (14)	0.0564 (13)	0.0352 (11)	0.0064 (11)	0.0016 (10)	−0.0011 (9)
C22	0.134 (3)	0.0563 (15)	0.0468 (14)	−0.0004 (15)	0.0242 (15)	−0.0084 (11)

### Geometric parameters (Å, °)

**Table:**

S1—C15	1.7033 (18)	C10—H10	0.9300
O2—C12	1.420 (2)	C11—C12	1.516 (3)
O2—H2	0.87 (3)	C12—C13	1.531 (3)
O3—C19	1.376 (2)	C13—C14	1.513 (3)
O3—C22	1.415 (3)	C13—H13A	1.00 (2)
N4—C15	1.321 (2)	C13—H13B	0.96 (2)
N4—C14	1.469 (2)	C14—C16	1.513 (3)
N4—H4	0.8600	C14—H14	1.029 (19)
N5—C15	1.340 (2)	C16—C17	1.377 (3)
N5—C12	1.456 (2)	C16—C21	1.382 (3)
N5—H5	0.8600	C17—C18	1.390 (3)
C6—C11	1.383 (3)	C17—H17	0.9300
C6—C7	1.386 (4)	C18—C19	1.381 (3)
C6—H6	0.9300	C18—H18	0.9300
C7—C8	1.340 (4)	C19—C20	1.378 (3)
C7—H7	0.9300	C20—C21	1.379 (3)
C8—C9	1.374 (5)	C20—H20	0.9300
C8—H8	0.9300	C21—H21	0.9300
C9—C10	1.393 (4)	C22—H22A	0.9600
C9—H9	0.9300	C22—H22B	0.9600
C10—C11	1.373 (3)	C22—H22C	0.9600
			
C12—O2—H2	113.2 (17)	C12—C13—H13B	108.1 (12)
C19—O3—C22	118.80 (17)	H13A—C13—H13B	109.9 (17)
C15—N4—C14	124.10 (16)	N4—C14—C16	109.86 (15)
C15—N4—H4	117.9	N4—C14—C13	106.90 (16)
C14—N4—H4	117.9	C16—C14—C13	116.45 (17)
C15—N5—C12	125.57 (15)	N4—C14—H14	107.9 (10)
C15—N5—H5	117.2	C16—C14—H14	107.9 (11)
C12—N5—H5	117.2	C13—C14—H14	107.6 (11)
C11—C6—C7	120.6 (3)	N4—C15—N5	118.50 (16)
C11—C6—H6	119.7	N4—C15—S1	121.63 (14)
C7—C6—H6	119.7	N5—C15—S1	119.87 (13)
C8—C7—C6	121.1 (3)	C17—C16—C21	118.15 (18)
C8—C7—H7	119.4	C17—C16—C14	123.44 (17)
C6—C7—H7	119.4	C21—C16—C14	118.37 (19)
C7—C8—C9	119.6 (3)	C16—C17—C18	121.58 (18)
C7—C8—H8	120.2	C16—C17—H17	119.2
C9—C8—H8	120.2	C18—C17—H17	119.2
C8—C9—C10	120.0 (3)	C19—C18—C17	119.23 (19)
C8—C9—H9	120.0	C19—C18—H18	120.4
C10—C9—H9	120.0	C17—C18—H18	120.4
C11—C10—C9	120.7 (3)	O3—C19—C20	115.45 (18)
C11—C10—H10	119.6	O3—C19—C18	124.77 (19)
C9—C10—H10	119.6	C20—C19—C18	119.79 (18)
C10—C11—C6	118.0 (2)	C19—C20—C21	120.2 (2)
C10—C11—C12	121.35 (19)	C19—C20—H20	119.9
C6—C11—C12	120.7 (2)	C21—C20—H20	119.9
O2—C12—N5	109.84 (16)	C20—C21—C16	121.1 (2)
O2—C12—C11	106.63 (16)	C20—C21—H21	119.5
N5—C12—C11	109.27 (15)	C16—C21—H21	119.5
O2—C12—C13	111.57 (17)	O3—C22—H22A	109.5
N5—C12—C13	108.21 (15)	O3—C22—H22B	109.5
C11—C12—C13	111.30 (16)	H22A—C22—H22B	109.5
C14—C13—C12	110.72 (17)	O3—C22—H22C	109.5
C14—C13—H13A	108.1 (12)	H22A—C22—H22C	109.5
C12—C13—H13A	110.4 (12)	H22B—C22—H22C	109.5
C14—C13—H13B	109.6 (12)		
			
C11—C6—C7—C8	−0.2 (4)	C12—C13—C14—N4	56.3 (2)
C6—C7—C8—C9	−0.5 (5)	C12—C13—C14—C16	179.46 (17)
C7—C8—C9—C10	0.5 (5)	C14—N4—C15—N5	4.5 (3)
C8—C9—C10—C11	0.1 (4)	C14—N4—C15—S1	−174.57 (15)
C9—C10—C11—C6	−0.8 (4)	C12—N5—C15—N4	3.2 (3)
C9—C10—C11—C12	−179.5 (2)	C12—N5—C15—S1	−177.72 (15)
C7—C6—C11—C10	0.9 (4)	N4—C14—C16—C17	69.8 (2)
C7—C6—C11—C12	179.6 (2)	C13—C14—C16—C17	−51.9 (3)
C15—N5—C12—O2	−101.5 (2)	N4—C14—C16—C21	−107.7 (2)
C15—N5—C12—C11	141.84 (19)	C13—C14—C16—C21	130.6 (2)
C15—N5—C12—C13	20.5 (3)	C21—C16—C17—C18	−1.7 (3)
C10—C11—C12—O2	−161.1 (2)	C14—C16—C17—C18	−179.17 (18)
C6—C11—C12—O2	20.3 (2)	C16—C17—C18—C19	0.4 (3)
C10—C11—C12—N5	−42.4 (3)	C22—O3—C19—C20	175.4 (2)
C6—C11—C12—N5	138.9 (2)	C22—O3—C19—C18	−4.4 (3)
C10—C11—C12—C13	77.0 (3)	C17—C18—C19—O3	−179.22 (18)
C6—C11—C12—C13	−101.6 (2)	C17—C18—C19—C20	1.0 (3)
O2—C12—C13—C14	70.8 (2)	O3—C19—C20—C21	179.17 (19)
N5—C12—C13—C14	−50.2 (2)	C18—C19—C20—C21	−1.0 (3)
C11—C12—C13—C14	−170.27 (18)	C19—C20—C21—C16	−0.3 (3)
C15—N4—C14—C16	−161.66 (18)	C17—C16—C21—C20	1.6 (3)
C15—N4—C14—C13	−34.5 (3)	C14—C16—C21—C20	179.26 (19)

### Hydrogen-bond geometry (Å, °)

**Table:**

*D*—H···*A*	*D*—H	H···*A*	*D*···*A*	*D*—H···*A*
O2—H2···S1^i^	0.88 (3)	2.51 (3)	3.3688 (18)	169 (2)
C14—H14···S1^i^	1.030 (19)	2.695 (18)	3.666 (2)	157.0 (14)
N4—H4···S1^ii^	0.86	2.47	3.2990 (19)	163
N5—H5···O3^iii^	0.86	2.18	3.032 (2)	169
C20—H20···S1^iv^	0.93	2.86	3.772 (2)	166
C6—H6···O2	0.93	2.33	2.671 (3)	101

Symmetry codes: (i) −*x*+1, −*y*+1, −*z*+1; (ii) −*x*+1, −*y*+2, −*z*+1; (iii) *x*, −*y*+3/2, *z*−1/2; (iv) *x*, −*y*+3/2, *z*+1/2.
